# Hypomethylating agents alone or in combination with venetoclax in very elderly acute myeloid leukemia patients: less treatment, better care?

**DOI:** 10.1007/s00277-026-06737-3

**Published:** 2026-01-11

**Authors:** Francesco Tarantini, Corinne Contento, Ernesto Vigna, Vera Carluccio, Giuseppina Greco, Crescenza Pasciolla, Lucia Ciuffreda, Giovanni Rossi, Marina Aurora Urbano, Alessandro D’Ambrosio, Lara Aprile, Vito Pier Gagliardi, Mario Delia, Immacolata Attolico, Paola Carluccio, Vincenzo Federico, Antonella Bruzzese, Nicola Di Renzo, Massimo Gentile, Giuseppe Tarantini, Anna Mele, Attilio Guarini, Lorella Maria Antonia Melillo, Angelo Michele Carella, Domenico Pastore, Ferdinando Frigeri, Alessandro Maggi, Cosimo Cumbo, Giorgina Specchia, Pellegrino Musto, Francesco Albano

**Affiliations:** 1 Department of Precision and Regenerative Medicine and Ionian Area - DiMePRe-J - Azienda Ospedaliero Universitaria Consorziale Policlinico - Hematology and Stem Cell Transplantation Unit, University of Bari “Aldo Moro”, P.zza G. Cesare, 11, 70124 Bari, Italy; 2 Hematology and Stem Cell Transplant Unit, “Vito Fazzi” Hospital, 73100 Lecce, Italy; 3 Department of Onco-Hematology, Hematology Unit, Cosenza, Italy; 4 Hematology and Transplant Unit, Dimiccoli Hospital, 76121 Barletta, BT Italy; 5 Hematology and Transplant Unit, Cardinal Panico Hospital, 73039 Tricase, Italy; 6 U.O. Di Ematologia, IRCCS Istituto Tumori Giovanni Paolo II, Bari, Italy; 7 Hematology and Transplant Unit, Policlinico Foggia Ospedaliero-Universitario, Foggia, Italy; 8 Hematology, Ospedale Casa Sollievo Della Sofferenza, San Giovanni Rotondo, Foggia, Italy; 9 Hematology Unit, A. Perrino Hospital, 72100 Brindisi, Italy; 10 AORN S. Anna E S. Sebastiano, Caserta, Italy; 11 Hematology Unit, Department of Hematology-Oncology, Moscati Hospital, 74010 Taranto, Italy; 12 School of Medicine, University of Bari “Aldo Moro”, 70124 Bari, Italy

**Keywords:** Hypomethylating agents, Venetoclax, Elderly acute myeloid leukemia, QoL

## Abstract

Hypomethylating agents (HMA) alone or in combination with venetoclax (VEN) are a mainstay for disease control in elderly acute myeloid leukemia (AML). We evaluated the non-inferiority of HMA monotherapy compared to HMA/VEN combination in 227 AML patients aged ≥ 75 years receiving HMA or HMA/VEN combination. No difference in overall survival (OS) was observed between the two groups, with HMA monotherapy demonstrating statistical non-inferiority. HMA-treated patients with favorable performance status had longer OS. The HMA/VEN group experienced higher mortality and worse QoL. HMA monotherapy offers comparable survival outcomes to HMA/VEN with reduced toxicity in elderly AML patients.

**Supplementary Information:**

The online version contains supplementary material available at 10.1007/s00277-026-06737-3.

Acute myeloid leukemia (AML) is a clonal hematopoietic malignancy characterized by the uncontrolled proliferation of immature myeloid cells in the bone marrow, blood, and other tissues, leading to hematopoietic failure. Predominantly affecting older adults, AML presents unique therapeutic challenges, particularly in patients non-eligible to intensive treatment, due to their limited tolerance to chemotherapy and increased comorbidities [1]. Over the past decade, hypomethylating agents (HMA) such as azacitidine (AZA) and decitabine (DEC) have emerged as treatment options for these patients, offering a less intensive approach by inducing leukemic cell apoptosis and differentiation through epigenetic modulation. This results in disease stabilization and transient control, particularly suited for elderly patients who are unable to tolerate more aggressive therapies [2, 3].

Recent treatment advances have incorporated venetoclax (VEN), a selective BCL-2 inhibitor that synergizes with HMA to enhance leukemic cell death. The VIALE-A study established the efficacy of AZA plus VEN in older (61% of patients aged ≥ 75 years), unfit AML patients, demonstrating a significant improvement in overall survival (OS), relapse-free survival and a higher complete remission (CR) rate compared to AZA alone [4]. Given these promising outcomes, VEN plus HMA has rapidly become a cornerstone of treatment in this setting, as emphasized in the 2022 European LeukemiaNet (ELN) guidelines. Notably, as emerged from recent real-world data, the ability of this combination to achieve deep and sustained remissions has made it a potential bridge to allogeneic hematopoietic stem cell transplantation (allo-HSCT) for patients who become eligible, underscoring the importance of pursuing CR in selected elderly AML patients [5].

Despite these advances, however, the addition of VEN is associated with increased toxicity risks that can impact treatment tolerability, particularly in real-world settings [6]. Prolonged cytopenia, especially neutropenia, are commonly observed and pose a significant risk for life-threatening infections, which can substantially compromise quality of life (QoL) in frail patients. Additionally, concerns about VEN-induced cardiotoxicity add complexity, especially for older patients with pre-existing cardiac comorbidities [7]. Moreover, a systematic review and meta-analysis of real-world data suggests lower survival rates compared to VIALE-A [8, 9]. In this context, the more intensive remission-targeted approach may not always align with the needs and goals of AML patients aged ≥ 75 years, where QoL and disease control may be prioritized over aggressive treatment.

Given these considerations, HMA monotherapy—though associated with lower CR rates—remains a viable therapeutic option in this older cohort. For AML patients ≥ 75 years who may be particularly vulnerable to treatment-related toxicities, a less intensive regimen aimed at controlling disease while minimizing adverse effects may be more appropriate than remission-targeted therapy [10].

This retrospective, observational, multicentre study aims to assess the non-inferiority of HMA monotherapy compared to the HMA/VEN combination in AML patients aged ≥ 75 years, providing real-world data on the comparative benefits of these regimens in terms of efficacy and QoL.

A total of 227 elderly (≥ 75 years at diagnosis) AML patients treated between 2018 and 2023 were enrolled in this study. A detailed description of patients enrolment criteria is reported in Supplementary File 1 and main patients clinical data are summarized in Table 1.

**Table 1:** Patients main biological and clinical characteristics

	HMA (*N* = 124)	HMA/VEN (*N* = 103)	*p*
Treatment type (%)	AZA = 94 DEC = 30 (76/24)	AZA/VEN = 80 DEC/VEN = 23 (78/22)	*NA*
Sex, M/F (%)	75/49 (60/40)	66/37 (64/36)	*0.68*
Median age, years (range)	79 (75–92)	77 (75–89)	***0.0004***
AML type: de novo/secondary/therapy related (%)	90/25/9 (73/20/7)	71/20/12 (69/19/12)	*0.52*
Median WBC/uL (range)	4045 (165–197630)	4100 (390–174660)	*0.77*
Median Hb g/dL (range)	8,7 (4,8–12,7)	8,4 (3,5–13,8)	*0.09*
Median PLT/uL (range)	49900 (5000–864000)	58000 (2000–290000)	*0.77*
Median BM blasts % (range)	40 (21–90)	40 (20–99)	*0.76*
ELN 2022 risk: low/intermediate/high (%)*	10/5/35 (20/10/70)	5/4/33 (12/10/78)	*0.56*
*NPM1* (A,B,D) positive/negative (%)*	12/84 (13/87)	9/79 (10/90)	*0.23*
*FLT3*-ITD positive/negative (%)*	8/88 (8/92)	18/70 (20/80)	***0.018***
ECOG-PS 0–2/3–4 (%)	68/56 (55/45)	98/5 (95/5)	** < *0.0001***
Hospitalization during first treatment cycle Yes/No (%)	89/35 (72/28)	91/12 (88/12)	***0.0037***
Median length of hospital stay in days (range)	12 (2–90)	13 (4–64)	*0.68*
Median N of outpatients visits per month (range)	3 (1–15)	4 (1–15)	***0.002***
Median *N* cycles of treatment (range)	7 (1–84)	4 (1–53)	***0.002***
CR/PR/NR (%)	54/21/49 (43,5/17/39,5)	46/10/47 (45/10/45)	*0.26*
N death events at 3 months from therapy initiation (%)	20/124 (16%)	25/103 (24%)	*0.13*
N death events at 6 months from therapy initiation (%)	32/124 (26%)	44/103 (43%)	***0.011***
Posaconazole prophylaxis Yes/No (%)	4/120 (5/95)	88/15 (85/15)	** < *0.001***
VEN 1st cycle schedule, 21/28 days (%)	NA	17/86 (17/83)	*NA*

*HMA* hypomethylating agents, *VEN* venetoclax, *AZA* azacitidine, *DEC* decitabine, *NA* not available, *AML* acute myeloid leukemia, *WBC* white blood cells, *Hb* haemoglobin, *PLT* platelets, *BM* bone marrow, *ELN* European leukemianet, *ECOG-PS* eastern cooperative oncology group performance status, *CR* complete remission, *PR* partial remission, *NR* no remission. *NA* not available. *Data not available for all patients enrolled

The local Ethics Committee of “Azienda Ospedaliero Universitaria Policlinico di Bari” approved the study (n.7896/2025). Informed consent was obtained from all patients before study inclusion, in accordance with the Declaration of Helsinki. Patients' records/information were anonymized and de-identified before analysis.

In detail, 124 out of 227 (55%) cases were treated with HMA alone: AZA (94/227, 42%) or DEC (30/227, 13%). On the contrary, 103/227 (45%) received HMA in combination with VEN: AZA/VEN (80/227, 35%) or DEC/VEN (23/227, 10%) (Table 1).

Median age at diagnosis was significantly lower in the HMA/VEN group compared to HMA (77 vs 79 years; *p* = 0.0004) and a higher proportion of patients in the HMA/VEN subset had favourable performance status [11] [(ECOG-PS 0–2 in 98/103 (95%) vs 68/124 (55%) in the HMA group; *p* < 0.0001]. Both cohorts had similar distribution in AML subtypes and ELN 2022 risk categories and no other significant differences emerged comparing main clinical data between the two groups (Table 1). By reverse-censoring, the median follow-up was 36 months across the entire cohort of patients (in detail, 60 months in the HMA arm and 15 months in the HMA/VEN arm). Notably, no difference exists between HMA and HMA/VEN groups in terms of OS (12 vs 8 months, Log-rank = 0.087 - Fig. 1A). In the accompanying Cox model, the hazard ratio (HR) for HMA alone was 0.76 (95%CI 0.56–1.05). When benchmarked against the prespecified non-inferiority margin of 25% (upper HR = 1.25), the entire confidence interval remained below the boundary, confirming that HMA monotherapy is statistically non-inferior to the combination regimen within this cohort. Interestingly, even when focusing on the subgroup of high-risk cases (ELN 2022), the OS analysis produced similar results between groups (8 vs 5 months, Log-rank = 0.38 – Fig. 1B). Moreover, the 6-month mortality rate was significantly higher in the HMA/VEN group [44/103 (43%) vs 32/124 (26%); *p* = 0.011) (Table 1).

**Fig. 1:**
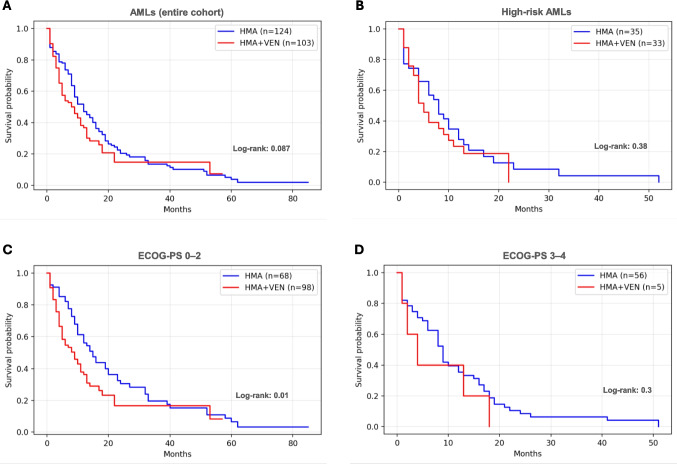
Survival analyses. Overall survival (OS) analyses between the two groups of patients (HMA vs HMA/VEN) for (**A**) the entire cohort enrolled; (**B**) the subgroup of high-risk cases (ELN 2022); (**C**) the subset of patients with a favourable performance status (ECOG-PS 0–2); (**D**) the subset of patients with a worse performance status (ECOG-PS 3–4)

Furthermore, considering the subset of patients with a favourable performance status (ECOG-PS 0–2), the OS of HMA group was significantly higher than HMA/VEN one (15 vs 9 months, Log-rank = 0.01 – Fig. 1C). No difference emerged for cases with an ECOG-PS 3–4, probably due to the rarity of cases analysed (Fig. 1D). The central role of patient performance status emerged as an independent predictor of OS from the univariable Cox proportional-hazards analysis (Supplementary File 1). We first performed univariable Cox regressions for each candidate prognostic factor; only variables with a *p*-value < 0.10 in univariable analysis were entered into the multivariable Cox model. In univariable analyses, VEN exposure showed a non-significant association with OS (HR 1.31, 95% CI 0.96–1.80, *p* = 0.0922), whereas ECOG was strongly associated with worse survival per one-point increase (HR 1.43, 95% CI 1.24–1.66, *p* < 0.0001). In the multivariable Cox model, which included VEN and ECOG as covariates based on the *p *< 0.10 criterion, VEN exposure was significantly associated with OS (HR 2.13, 95% CI 1.49–3.06, *p* < 0.0001), and ECOG remained an independent predictor of poorer survival (HR 1.66 per point, 95% CI 1.41–1.96, *p* < 0.0001).

Age, secondary AML, and *FLT3* mutations (ITD or TKD) did not meaningfully influence outcome, and *NPM1* mutation was associated with a lower—but non-significant—hazard (HR = 0.64; 95%CI 0.36–1.15; *p* = 0.13) (Supplementary File 1). Regarding the occurrence of *NPM1* and *FLT3*-ITD variants, no difference was observed between the groups for *NPM1*; however, the occurrence of *FLT3*-ITD was higher in the HMA/VEN group compared to the HMA group (18/88, 20% vs 8/96, 8%; *p* = 0.018 – Table 1]. To rule out any potential influence of *FLT3* mutational status on the OS of the two groups, a dedicated analysis was performed on cases for which this information was available (see Table 1). No significant difference emerged between the groups (9 vs. 10 months, log-rank = 0.828; see Supplementary File 1). Hospitalization during the first treatment cycle occurred more frequently in the HMA/VEN than in HMA group (91/103, 88% vs 89/124, 72%; *p* = 0.0037), although the median hospital stay (meaning the number of days spent inward during the first cycle of treatment) was comparable (13 vs 12 days, *p* = 0.68) (Table 1). Moreover, median outpatient visits per month (excluding those for therapy administration) were higher in the HMA/VEN group compared to HMA (4 vs 3; *p* = 0.002) even if the median number of treatment cycles was significantly lower in HMA/VEN patients than in HMA (4 vs 7; *p* = 0.002), possibly due to early toxicity (Table 1). Posaconazole prophylaxis was performed in the majority of patients in the HMA/VEN arm (88/103, 85%). Accordingly, in 74/88 patients, VEN dose was reduced to 100 mg/day, while in 11/88 VEN was administered at 50 mg/day. Two patients received VEN 200 mg/day and only one patient 400 mg/day.

The AML treatment is rapidly changing, turning into an individualized approach on the basis of the ever-expanding amount of biological and clinical information, and the advent of new drugs. In this context, the HMA/VEN combination plays a pivotal role for all patients deemed unfit for intensive programs. Nevertheless, the extraordinary VEN efficacy seems to go far beyond this population; evidence demonstrates its effectiveness in the induction phase in combination with intensive chemotherapy, as a bridging therapy to transplant procedure, in the context of *NPM1* molecular relapse, and even in relapsed/refractory disease [12–15]. On the other hand, real-world data in the elderly AML population seem to suggest that all this power comes at a cost.

Accordingly, our data seem to suggest that in the very elderly AML population, the HMA therapy is not inferior to HMA/VEN combination in terms of OS. Moreover, patients with an ECOG-PS 0–2 seem to significantly benefit from the HMA monotherapy. These observations, however, must be carefully considered due to the retrospective nature of the study. We may argue that the combination of a less toxic, non-intensive, therapy with a better baseline PS could result in the best possible treatment offer, considering that for the majority of these patients a prolonged OS together with the preservation of QoL would be the reasonable goals of treatment. In line with this concept, the 6 months mortality in the HMA/VEN arm is higher than that expected from the VIALE-A data, with a substantial difference in favor of HMA monotherapy: we may argue that this discrepancy reflects the complexity of disease burden and real-life management of these patients, including VEN dosing; up-to-date evidence demonstrates, in fact, that it should be safely adapted to less toxic schedules without affecting its efficacy [16]. One of the limitations of our study is the scarcity of data regarding the molecular characterization of patients. This is likely a reflection of real-life clinical practice, where molecular profiling is more commonly reserved for younger patients, typically below the age threshold of 75 years. Nowadays, the availability of targeted drugs will change this approach towards a thorough biological characterization [17].

In this scenario, a more extended use of VEN aside the elderly unfit AML population seems to be a key point. Further, prospective studies will clarify this open question: are we heading into a three (intensive, less-intensive, non-intensive) routes of treatment options for AML?

## Supplementary Information

Below is the link to the electronic supplementary material.

Supplementary file1 (DOCX 76 KB)

## Data Availability

Data is available upon request.

## Abbreviations

AML: Acute myeloid leukemia
HMA: Hypomethylating agents
AZA: Azacitidine
DEC: Decitabine
VEN: Venetoclax
OS: Overall survival
CR: Complete remission
ELN: European LeukemiaNet
Allo-HSCT: Allogeneic hematopoietic stem cell transplantation
QoL: Quality of life
HR: Hazard ratio
ECOG-PS: Eastern Cooperative Oncology Group Performance Status
