# Supplementary material for: Hypomethylating agents alone or in combination with venetoclax in very elderly acute myeloid leukemia patients: less treatment, better care?
Source: Ann Hematol. 2026 Jan 11;105(1):1. doi: 10.1007/s00277-026-06737-3 (PMC12791063; doi:10.1007/s00277-026-06737-3)
Supplement: Supplementary file 1 — Supplementary file1 (DOCX 76 KB) [file 277_2026_6737_MOESM1_ESM.docx]

**Supplementary File 1**

**1. Methods**

**1.1. Criteria for patient selection**

***1.1.1. Inclusion criteria***

- Age ≥75 years at the time of diagnosis.
- Diagnosis of acute myeloid leukemia (AML) according to WHO criteria [1, 2].
- Treatment with azacitidine or decitabine as monotherapy or in combination with venetoclax in a clinical setting.
- Available clinical, laboratory, and treatment data sufficient for the evaluation of primary and secondary endpoints.

***1.1.2. Exclusion criteria***

- Concomitant diagnosis of other active untreated hematologic or solid malignancies.
- Diagnosis of myeloproliferative neoplasm in blast phase.
- Prior antineoplastic treatments for AML.
- Intensive induction therapy or stem cell transplantation.

**1.2. Statistical analysis**

Baseline characteristics were summarized for each treatment cohort. Continuous variables were reported as median (inter-quartile range, IQR) and compared with the Mann–Whitney U-test; categorical variables were expressed as counts and proportions and compared with Pearson’s χ² test or Fisher’s exact test (when appropriate).

Early mortality was assessed at 3 and 6 months. Cumulative death counts were tabulated for each arm, and absolute risks, risk differences and risk ratios (RRs) with 95 % CIs were computed. Overall survival (OS) was defined as the interval between date of diagnosis and date of death from any cause or last follow-up, and was analysed with the Kaplan–Meier method and compared using the log-rank test. The primary comparative analysis used a Cox proportional-hazards model with a single binary covariate that coded hypomethylating-agent (HMA) monotherapy as 1 and HMA + venetoclax (HMA + VEN) as 0. Results are reported as hazard ratios (HRs) with two-sided 95 % confidence intervals (CIs).

Non-inferiority of HMA alone was assessed against a prespecified margin of 25 %, expressed as an upper HR boundary of 1.25. HMA was judged non-inferior if the upper limit of its 95 % CI lay entirely below 1.25. This margin reflects commonly accepted thresholds in hematological trials and balances potential efficacy loss against the toxicity, cost and myelosuppression associated with VEN. The proportional-hazards assumption was verified with Schoenfeld residuals. Observations missing OS or event status were excluded case-wise; data completeness exceeded 90%.

Covariates potentially associated with OS were first screened in univariable Cox proportional-hazards models; results are reported as hazard ratios (HR) with 95 % confidence intervals (CI). Only variables with a p-value <0.10 in univariable analysis were subsequently entered into the multivariable Cox model. The candidate covariates included age at diagnosis (continuous), ECOG performance status (ordinal), disease type (de novo vs secondary/therapy-related AML), *NPM1* mutation, *FLT3*-ITD mutation, *FLT3*-TKD mutation, and exposure to VEN during induction. Variables with fewer than two levels after data cleaning were excluded. The proportional hazards assumption was assessed by Schoenfeld residuals. Missing data were handled by case-wise deletion for the specific model in which the variable appeared; no variable missed more than 10% of observations.

All analyses were performed with R version 4.3 (packages survival, survminer, epitools) and verified in Python 3.10 (lifelines, statsmodels, SciPy). Statistical significance was set at two-sided p < 0.05.

**2. Supplementary results**

**2.1. Univariate Cox analysis**

| **Variable** | **HR** | **CI_lower** | **CI_upper** | **p value** |
| --- | --- | --- | --- | --- |
| *NPM1* mut | 0,642686 | 0,360578 | 1,145508 | 0,1338 |
| Secondary AML | 1,024146 | 0,735519 | 1,426035 | 0,8876 |
| Age | 1,030445 | 0,986409 | 1,076448 | 0,1783 |
| *FLT3*-TKD mut | 1,123939 | 0,49379 | 2,558249 | 0,7806 |
| Venetoclax | 1,311962 | 0,956473 | 1,799574 | 0,0921 |
| *FLT3*-ITD mut | 1,384447 | 0,854609 | 2,242772 | 0,1862 |
| ECOG PS | 1,434058 | 1,240598 | 1,657686 | <0,0001 |

**2.2. Multivariate Cox analysis**

| **Variable** | **HR** | **CI_lower** | **CI_upper** | **p value** |
| --- | --- | --- | --- | --- |
| Venetoclax | 2,13045582 | 1,48530833 | 3,055824775 | <0,0001 |
| ECOG PS | 1,66186378 | 1,40884224 | 1,960326811 | <0,0001 |

**2.3 Survival Analysis**

Only cases for which the *FLT3* mutational status was available, were included.

**
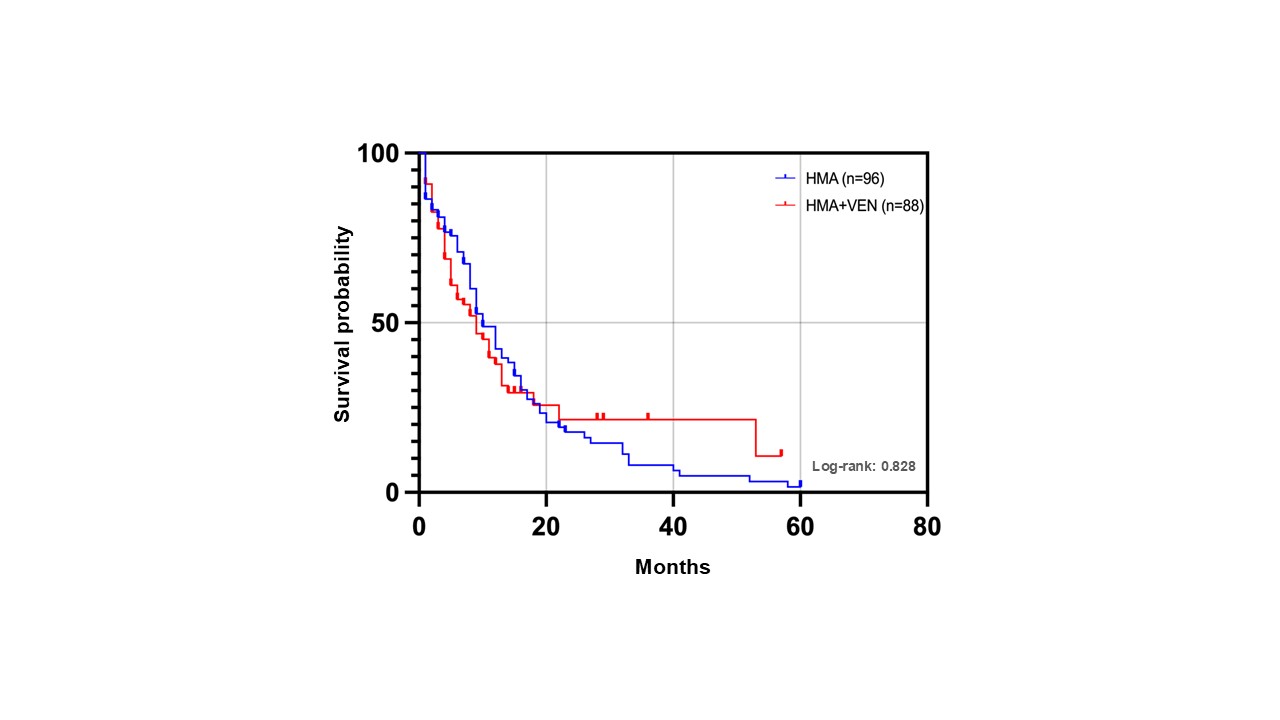
**

**3. References**

1. Khoury JD, Solary E, Abla O, et al (2022) The 5th edition of the World Health Organization Classification of Haematolymphoid Tumours: Myeloid and Histiocytic/Dendritic Neoplasms. Leukemia 36:1703–1719. https://doi.org/10.1038/s41375-022-01613-1

2. Arber DA, Orazi A, Hasserjian R, et al (2016) The 2016 revision to the World Health Organization classification of myeloid neoplasms and acute leukemia. Blood 127:2391–2405. https://doi.org/10.1182/blood-2016-03-643544
